# Maternal smoking during pregnancy in relation to adolescent anxiety and poor mental health: A cross-sectional study based on NHANES 2007–2012

**DOI:** 10.18332/tid/226577

**Published:** 2026-09-12

**Authors:** Ya Liu, Jiaqi Deng, Yong Zhang

**Affiliations:** 1 Department of Nursing, The First Affiliated Hospital of Xi'an Jiaotong University, Xi'an, China; 2 College of Medicine & Forensics, Xi'an Jiaotong University Health Science Center, Xi'an, China; 3 School of Nursing, Southwest Medical University, Luzhou, China; 4 Department of Radiation Oncology, The First Affiliated Hospital of Xi'an Jiaotong University, Xi'an, China

**Keywords:** maternal smoking during pregnancy, adolescent mental health, anxiety, poor mental health, NHANES

## Abstract

**INTRODUCTION:**

Maternal smoking during pregnancy (MSDP) has been suggested as a potential contributor to adolescent mental health problems, yet existing evidence remains inconsistent. This study aimed to examine the association between MSDP and anxiety and poor mental health outcomes among US adolescents.

**METHODS:**

This is a secondary analysis of pooled cross-sectional data from the National Health and Nutrition Examination Survey (NHANES) 2007–2012, which employs a complex, multistage probability sampling design to ensure national representativeness. A total of 1445 adolescents aged 12–15 years were included. MSDP was assessed via parental report. Anxiety and poor mental health symptoms were measured by self-reported numbers of days feeling anxious or mentally unwell in the past 30 days. Weighted negative binomial regression and logistic regression models were used to estimate the rate ratio (RR) and odds ratio (OR), accounting for the complex survey design. Sensitivity analyses were performed using unweighted logistic regression models.

**RESULTS:**

Of the participants, 14.9% were exposed to MSDP. The prevalence of high anxiety (23.2% vs 12.4%, p=0.002) and poor mental health (28.2% vs 12.3%, p=0.001) was greater in the MSDP-exposed group than in the unexposed group. After full adjustment, MSDP was associated with a 1.68-fold increase in the number of anxiety days (95% CI: 1.19–2.37) and a 1.97-fold increase in the number of poor mental health days (95% CI: 1.40–2.77). As categorical outcomes, MSDP was also associated with higher odds of severe anxiety (adjusted odds ratio, AOR=3.03; 95% CI: 1.45–6.29) and severe poor mental health (AOR=3.11; 95% CI: 1.55–6.24). Sensitivity analyses confirmed the robustness of these associations.

**CONCLUSIONS:**

In this cross-sectional study, MSDP was associated with a higher likelihood of anxiety and poor mental health in adolescents. Given the study design, causality cannot be established. Future longitudinal studies are warranted to confirm these findings and explore potential underlying mechanisms.

## INTRODUCTION

Adolescence is a crucial developmental stage during which mental health disorders, particularly anxiety and depression, commonly manifest^1^. Epidemiological data indicate that approximately 11.6% of adolescents are affected by anxiety disorders, while 12.9% experience depressive disorders^2,3^. The prevalence of these conditions rises substantially by early adolescence: by the age of 14 years, approximately 38% of adolescents exhibit symptoms of anxiety, and 3.1% are clinically diagnosed with depression^4,5^. As a major global health burden, adolescent mental health requires attention, yet the prevalence and burden of anxiety and depression have shown minimal decline over recent decades^1,6^.

Maternal smoking during pregnancy (MSDP) is a well-established modifiable risk factor associated with various adverse health outcomes in offspring^7-9^. Despite known risks, MSDP remains prevalent, with approximately 25% of pregnant women in Western countries continuing to smoke, with over 70% of these being daily users^10^. Although a number of studies have linked MSDP to offspring internalizing behaviors such as anxiety and depression, the evidence remains inconclusive. Some studies report that MSDP increases the likelihood of anxiety and depression in early adulthood by 45% and 75%, respectively^11^, while a large Norwegian cohort study found that the association was no longer significant by the age of 5 years^12^. Similarly, a propensity-score-matched analysis reported no significant association^13^. Furthermore, sibling studies that control for shared family environments have produced conflicting results^14-16^.

Given the preventability of MSDP, its association with adolescent mental health deserves further investigation using nationally representative data. This study uses pooled cross-sectional data from the National Health and Nutrition Examination Survey (NHANES) 2007–2012 to examine the relationship between MSDP and anxiety and poor mental health symptoms in US adolescents aged 12–15 years.

## METHODS

### Study design and population

This is a secondary analysis of pooled cross-sectional data from the National Health and Nutrition Examination Survey (NHANES) 2007–2012. NHANES is conducted by the National Center for Health Statistics (NCHS) to assess the health and nutritional status of the US non-institutionalized civilian population. The survey employs a complex, multistage probability sampling strategy to ensure national representativeness^17^. The study protocol was approved by the NCHS Research Ethics Review Board, and informed consent was obtained from all participants.

**Figure 1:**
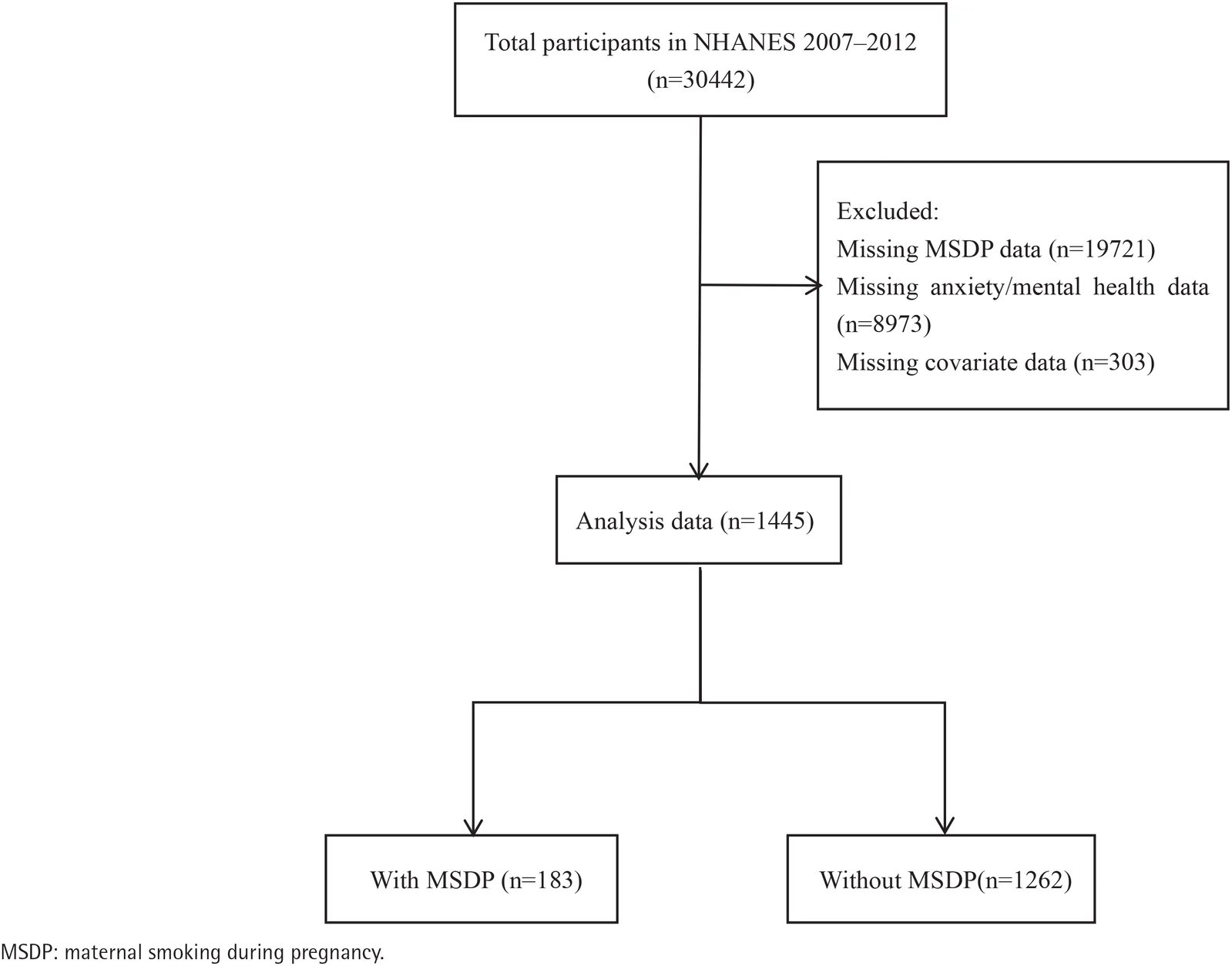
Flowchart of participant enrollment from NHANES 2007–2012

The study included adolescents aged 12–15 years whose mothers provided information on smoking during pregnancy and who had complete data on anxiety and mental health outcomes. Exclusion criteria were: 1) missing maternal smoking during pregnancy data (n=19721); 2) missing anxiety or mental health data (n=8973); and 3) missing covariate data (n=303). A total of 1445 participants were included in the final analysis (Figure 1).

### Maternal smoking during pregnancy (MSDP)

In the NHANES 2007–2012 dataset, the variable ‘Mother smoked when pregnant’ was assessed among participants aged 1–15 years through a questionnaire asking if their biological mother smoked at any time during her pregnancy with the participant. Responses affirming maternal smoking were classified as ‘MSDP’, while negative responses were classified as ‘no MSDP’.

### Anxiety and poor mental health assessment

Anxiety was assessed in private interviews with respondents aged ≥12 years, using the following question: ‘In the past 30 days, how many days have you felt worried, nervous, or anxious?’. Based on previous research methods, anxiety was categorized as low anxiety (0–6 days) or high anxiety (7–30 days)^17^. Mental health was assessed with the following question: ‘Thinking back over the past 30 days, how was your mental health, including stress, depression, and emotional problems? How many days did you feel your mental health was poor?’. Similarly, individuals were categorized into better mental health (0–6 days of poor mental health) or poor mental health (7–30 days of poor mental health)^17^. To further rigorously define mental health status, a stricter criterion was used, with 15–30 days being classified as severe anxiety or severe poor mental health.

### Potential covariates

Covariates were selected a priori based on existing literature and included the following: age (categorical: 12, 13, 14, and 15 years), sex (male, female), race/ethnicity (Non-Hispanic White, Non-Hispanic Black, Other), BMI (categorized as <25, 25–29.9, and ≥30 kg/m^2^), family poverty income ratio (PIR; categorized as <1.3, 1.3–3.5, and ≥3.5), household size (categorized as ≤4 and ≥5), household head’s education level (<high school, high school, >high school), the adolescent’s education level (elementary school, middle school, high school), hypertension (yes, no), health insurance status (yes, no), routine healthcare access (yes, no), physical activity (inactive, moderate, vigorous), maternal age at birth (continuous, years), low birth weight (yes, no), parental marital status (married/cohabiting, never married, widowed/divorced/separated), and self-reported poor physical health (yes, no).

### Statistical analysis

To ensure that the study population was representative of the US population, we accounted for the complex sampling design and sampling weights in the analysis. Normality tests were conducted for continuous variables, with normally distributed data presented as means (standard errors), and skewed data presented as medians (interquartile ranges). Group comparisons were performed using Student’s t-test (for normally distributed data) or the Mann-Whitney U test (for skewed data). Categorical variables were expressed as weighted percentages, and comparisons were made using chi-squared tests.

To assess the relationship between maternal smoking during pregnancy (MSDP) and anxiety and poor mental health, we used the number of days participants felt anxious or had poor mental health in the past 30 days as count and categorical data, applying weighted negative binomial regression models and logistic regression models to calculate relative risk (RR) and odds ratio (OR). Three models were used in this study: Model 1: the unadjusted crude model; Model 2: adjusted for adolescent age, sex, race/ethnicity, family PIR, household size, household head’s education level, and adolescent’s education level; Model 3: the fully adjusted model, further adjusted for BMI, hypertension, physical activity, health insurance status, routine healthcare access, maternal age at birth, low birth weight, parental marital status, and self-reported poor physical health.

As a sensitivity analysis, unweighted logistic regression models were also performed to evaluate the robustness of the findings under an unweighted framework. Multicollinearity among covariates was assessed using variance inflation factors (VIF) based on an unweighted logistic regression model. All equivalent VIF values GVIF^(1/2df)^ were <5, indicating no serious multicollinearity in the fully adjusted models. All analyses were conducted using R version 4.2 (R Foundation for Statistical Computing, Vienna, Austria) with the survey package. A two-sided p<0.05 was considered statistically significant.

## RESULTS

### Baseline characteristics of participants

Table 1 presents the baseline characteristics of a total of 1445 adolescents aged 12–15 years from NHANES 2007–2012, stratified by MSDP. Approximately 14.9% of the adolescents were exposed to MSDP. Mothers in the MSDP group were generally younger compared with those in the no MSDP group (median: 25.0 vs 28.0 years, p<0.001). The proportion of low-birth-weight infants was higher in the MSDP group (19.2% vs 8.9%, p=0.004). Significant differences between groups were observed in race/ethnicity, family poverty income ratio (PIR), household head’s education level, and parental marital status (all p<0.05). No significant differences were found in adolescent age, sex, body mass index (BMI), hypertension, adolescent education level, household size, physical activity, access to routine healthcare, health insurance status, or self-reported poor physical health (all p>0.05).

**Table 1:** Baseline characteristics of US adolescents aged 12–15 years, stratified by maternal smoking during pregnancy (MSDP), from the National Health and Nutrition Examination Survey (NHANES) 2007–2012 (N=1445)

*Characteristics*	*Total (N=1445)* *n (%)*	*MSDP (N=183)* *n (%)*	*no MSDP (N=1262)* *n (%)*	*p*
**Mother’s age at child’s birth** (years), median (IQR)	27.00 (23.00–31.00)	25.00 (20.00–29.00)	28.00 (23.00–32.00)	<0.001
**Age** (years)				0.572
12	348 (23.9)	41 (22.2)	307 (24.1)	
13	366 (23.5)	48 (26.5)	318 (23.0)	
14	386 (26.8)	49 (22.9)	337 (27.4)	
15	345 (25.9)	45 (28.4)	300 (25.4)	
**Sex**				0.369
Male	747 (50.6)	91 (46.7)	656 (51.3)	
Female	698 (49.4)	92 (53.3)	606 (48.7)	
**Race/ethnicity**				<0.001
Non-Hispanic White	444 (60.4)	101 (78.6)	343 (57.2)	
Non-Hispanic Black	346 (13.0)	40 (9.9)	306 (13.5)	
Other race	655 (26.6)	42 (11.5)	613 (29.2)	
**Family PIR**				<0.001
<1.3	591 (28.3)	94 (39.7)	497 (26.3)	
1.3–3.5	529 (37.2)	71 (45.7)	458 (35.8)	
≥3.5	325 (34.4)	18 (14.6)	307 (37.9)	
**BMI** (kg/m^2^)				0.160
<25	1028 (73.7)	123 (65.3)	905 (75.2)	
25–29.9	239 (15.9)	33 (19.8)	206 (15.2)	
≥30	178 (10.4)	27 (15.0)	151 (9.6)	
**Hypertension**				0.977
No	1416 (98.3)	180 (98.3)	1236 (98.3)	
Yes	29 (1.7)	3 (1.7)	26 (1.7)	
**Education level**				0.578
Elementary school	137 (8.7)	19 (11.1)	118 (8.3)	
Middle school	1033 (71.5)	133 (68.9)	900 (72.0)	
High school	275 (19.7)	31 (20.0)	244 (19.7)	
**Household size**				0.128
≤4	727 (57.9)	105 (64.1)	622 (56.8)	
≥5	718 (42.1)	78 (35.9)	640 (43.2)	
**Household head’s education level**				0.005
<High school	403 (19.3)	53 (23.1)	350 (18.7)	
High school	314 (19.9)	46 (29.0)	268 (18.3)	
>High school	728 (60.8)	84 (47.9)	644 (63.0)	
**Parental marital status**				0.002
Married/living with partner	962 (73.3)	100 (61.0)	862 (75.4)	
Single/divorced/widowed	483 (26.7)	83 (39.0)	400 (24.6)	
**Physical activity**				0.550
Inactive	887 (55.5)	105 (54.6)	782 (55.6)	
Moderate	418 (32.8)	53 (31.1)	365 (33.2)	
Vigorous	140 (11.7)	25 (14.3)	115 (11.2)	
**Poor physical health**				0.317
No	1317 (91.4)	163 (89.1)	1154 (91.9)	
Yes	128 (8.6)	20 (10.9)	108 (8.1)	
**Routine place to go for healthcare**				0.506
Yes	1351 (95.0)	176 (96.5)	1175 (94.7)	
No	94 (5.0)	7 (3.5)	87 (5.3)	
**Health insurance status**				0.514
Yes	1277 (90.3)	162 (88.5)	1115 (90.6)	
No	168 (9.7)	21 (11.5)	147 (9.4)	
**Low birth weight**				0.004
No	1266 (89.6)	145 (80.8)	1121 (91.1)	
Yes	179 (10.4)	38 (19.2)	141 (8.9)	
**High anxiety**				0.002
No	1252 (86.0)	144 (76.8)	1108 (87.6)	
Yes	193 (14.0)	39 (23.2)	154 (12.4)	
**Poor mental health**				0.001
No	1269 (85.3)	142 (71.8)	1127 (87.7)	
Yes	176 (14.7)	41 (28.2)	135 (12.3)	

Values are presented as weighted percentages for categorical variables and weighted medians with interquartile range (IQR) for continuous variables, accounting for the complex survey design. P-values were calculated using the Rao-Scott χ² test for categorical variables and the Wald test for continuous variables. MSDP: maternal smoking during pregnancy. PIR: poverty income ratio. BMI: body mass index.

### Relationship between MSDP and offspring anxiety and poor mental health

As shown in Supplementary file Figure S1, among adolescents whose mothers had MSDP, 23.2% reported experiencing ≥7 days of anxiety in the past month, and 28.2% reported ≥7 days of poor mental health. In contrast, the corresponding percentages in the no MSDP group were 12.4% for both anxiety and poor mental health, with significant differences between the two groups (p=0.001). Furthermore, the difference between the two groups was even greater for ≥15 days of anxiety (13.6% vs 4.7%) and poor mental health (17.7% vs 5.5%).

Table 2 presents the association between MSDP and anxiety and poor mental health outcomes. In the weighted negative binomial regression models, MSDP was associated with 1.68 times the number of anxiety days (adjusted rate ratio, ARR=1.68; 95% CI: 1.19–2.37; p=0.003) and 1.97 times the number of poor mental health days (ARR=1.97; 95% CI: 1.40–2.77; p<0.001) in the fully adjusted Model 3. When the outcome variables were analyzed as binary outcomes using weighted logistic regression, MSDP was associated with higher odds of high anxiety (≥7 days) (AOR=1.82; 95% CI: 1.03–3.21; p=0.039) and poor mental health (≥7 days) (AOR=2.28; 95% CI: 1.30–4.00; p=0.004). When the threshold was increased to ≥15 days, MSDP was associated with higher odds of severe anxiety (AOR=3.03; 95% CI: 1.45–6.29; p=0.003) and severe poor mental health (AOR=3.11; 95% CI: 1.55–6.24; p =0.001).

**Table 2:** Weighted negative binomial regression and logistic regression models for the association between maternal smoking during pregnancy (MSDP) and anxiety and poor mental health outcomes among adolescents aged 12–15 years, NHANES 2007–2012 (N=1445)

*Variables*	*Model 1*		*Model 2*		*Model 3*	
	*RR (95% CI)*	*p*	*ARR (95% CI)*	*p*	*ARR (95% CI)*	*p*
Days of anxiety in the past 30 days	1.80 (1.28–2.52)	0.001	1.83 (1.28–2.62)	0.001	1.68 (1.19–2.37)	0.003
Days of poor mental health in the past 30 days	2.32 (1.69–3.20)	<0.001	2.12 (1.50–2.99)	<0.001	1.97 (1.40–2.77)	<0.001
	***OR (95% CI)***		***AOR (95% CI)***		***AOR (95% CI)***	
Higher anxiety (≥7 days)	2.14 (1.32–3.48)	0.002	2.09 (1.23–3.55)	0.006	1.82 (1.03–3.21)	0.039
Poor mental health (≥7 days)	2.79 (1.73–4.52)	<0.001	2.46 (1.43–4.24)	0.001	2.28 (1.30–4.00)	0.004
Severe anxiety (≥15 days)	3.22 (1.68–6.17)	<0.001	3.48 (1.76–6.87)	<0.001	3.03 (1.45–6.29)	0.003
Severe poor mental health (≥15 days)	3.72 (2.03–6.80)	<0.001	3.12 (1.60–6.12)	0.001	3.11 (1.55–6.24)	0.001

All models accounted for the complex survey design of NHANES 2007–2012 by applying sampling weights. Model 1: unadjusted. Model 2: adjusted for adolescent age, sex, race/ethnicity, family PIR, household size, household head’s education level, and adolescent education level. Model 3: adjusted as for Model 2 plus BMI, hypertension, physical activity, health insurance status, routine healthcare access, maternal age at birth, low birth weight, parental marital status, and self-reported poor physical health. The reference group is the ‘No MSDP’ group for all models. A two-sided p<0.05 was considered statistically significant. ARR: adjusted rate ratio. AOR: adjusted odds ratio. PIR: poverty income ratio. BMI: body mass index.

### Sensitivity analysis

When analyzing unweighted data and converting the outcome variables to categorical variables (with ≥7 days defining high anxiety and poor mental health), multivariable logistic regression analysis was performed (Table 3). In the fully adjusted Model 3, the odds of high anxiety (≥7 days) in the offspring of mothers with MSDP were 1.84 times those of the no MSDP group (AOR=1.84; 95% CI: 1.19–2.85; p=0.006), and the odds of poor mental health (≥7 days) were 2.01 times those of the no MSDP group (AOR=2.01; 95% CI: 1.29–3.12; p=0.002). When the threshold was increased (with ≥15 days defining severe anxiety and severe poor mental health), the results in Model 3 showed that the odds of severe anxiety (≥15 days) in the offspring of mothers with MSDP were 2.37 times higher than in the no MSDP group (AOR=2.37; 95% CI: 1.31–4.30; p=0.004), and the odds of severe poor mental health (≥15 days) were 2.61 times higher (AOR=2.61; 95% CI: 1.49–4.57; p=0.001). In the fully adjusted models, all equivalent VIF values were <5 (range: 1.01–1.31), indicating no serious multicollinearity among covariates(Supplementary file Table S1).

**Table 3:** Unweighted logistic regression sensitivity analysis for the association between maternal smoking during pregnancy (MSDP) and adolescent anxiety and poor mental health among adolescents aged 12–15 years, NHANES 2007–2012 (N=1445)

*Variables*	*Model 1*		*Model 2*		*Model 3*	
	*OR (95% CI)*	*p*	*AOR (95% CI)*	*p*	*AOR (95% CI)*	*p*
Higher anxiety (≥7 days)	1.95 (1.32–2.88)	0.001	1.91 (1.25–2.91)	0.003	1.84 (1.19–2.85)	0.006
Poor mental health (≥7 days)	2.41 (1.63–3.56)	<0.001	2.13 (1.39–3.26)	<0.001	2.01 (1.29–3.12)	0.002
Severe anxiety (≥15 days)	2.37 (1.40–4.03)	0.001	2.59 (1.47–4.57)	0.001	2.37 (1.31–4.30)	0.004
Severe poor mental health (≥15 days)	3.17 (1.93–5.20)	<0.001	2.68 (1.55–4.61)	<0.001	2.61 (1.49–4.57)	0.001

This sensitivity analysis was performed using unweighted logistic regression models. Model 1: unadjusted. Model 2: adjusted for adolescent age, sex, race/ethnicity, family PIR, household size, household head’s education level, and adolescent education level. Model 3: adjusted as for Model 2 plus BMI, hypertension, physical activity, health insurance status, routine healthcare access, maternal age at birth, low birth weight, parental marital status, and self-reported poor physical health. The reference group is the ‘No MSDP’ group. A two-sided p<0.05 was considered statistically significant. AOR: adjusted odds ratio. PIR: poverty income ratio. BMI: body mass index.

## DISCUSSION

In this study, using cross-sectional data from a nationally representative sample of US adolescents, the adjusted models revealed a significant positive association between MSDP and adolescent anxiety and poor mental health symptoms. A particularly noteworthy finding is that the odds of severe anxiety and severe poor mental health were substantially higher than those observed for the broader outcome definitions. Sensitivity analyses further confirmed the robustness of these associations, as the relationships remained significant even when unweighted data were used. Moreover, as the threshold for anxiety and poor mental health severity was raised, the corresponding effect sizes also increased.

The impact of MSDP on offspring emotional and behavioral problems has attracted significant attention, yet research findings remain controversial. Some studies report no association between MSDP and offspring internalizing behaviors^18-21^, with sibling studies controlling for shared family factors reaching similar conclusions^14,15^. These studies argue that the observed association may largely reflect family background confounders – such as young maternal age, low socioeconomic status, and maternal mental illness. Conversely, a large-scale prospective study (UK Biobank, n=502394) confirmed that MSDP was associated with a higher likelihood of anxiety (HR=1.11; 95% CI: 1.07–1.16) and depression (HR=1.19; 95% CI: 1.14–1.23)^22^. Studies with rigorous designs, including sibling studies controlling for offspring smoking and childhood adversity, have reported dose-dependent associations, with each additional pack/day of smoking during pregnancy associated with nearly twofold higher odds of adult depression (OR=1.93; 95% CI: 1.10–3.37)^16,23^. Furthermore, a two-sample Mendelian randomization (MR) study reported significant positive associations with the odds of anxiety (OR=1.03; 95% CI: 1.00–1.05) and severe depression (OR=1.92; 95% CI: 1.29–2.88)^24^. In conclusion, while family confounders cannot be ignored, evidence from well-controlled sibling and Mendelian randomization studies increasingly supports an independent, potentially dose-dependent effect of MSDP on offspring mental health.

Multiple interrelated biological pathways may collectively contribute to the association between MSDP and offspring mental health problems. First, nicotine readily crosses the placenta and blood-brain barrier, leading to the activation of nicotinic acetylcholine receptors (nAChRs) in the fetal brain and disruption of key neurotransmitter systems, including GABA, glutamate, and dopamine signaling^25-27^. Second, nicotine-induced placental vasoconstriction reduces blood flow and limits oxygen supply to the fetus, resulting in fetal hypoxia, which has been linked to anxiety and an increased likelihood of mental health disorders^17,28,29^. Moreover, MSDP has been associated with increased oxidative stress and the upregulation of inflammatory markers, both of which have been implicated in the development of mental health problems^30,31^. Furthermore, MSDP may disrupt the fetal hypothalamic-pituitary-adrenal (HPA) axis^32^, a critical system for stress response, and may lead to significant structural changes in offspring brain development, including reduced brain volume and impaired cortical formation, which could provide the underlying basis for the emotional and behavioral issues observed in exposed adolescents^33,34^. Finally, smoking behavior is genetically correlated with mental disorders such as depression^35^, and the effects of maternal smoking on offspring mental health may also occur through passive gene-environment correlations^36^.

### Limitations

This study has several limitations. First, MSDP was assessed via retrospective maternal self-report, which is susceptible to recall bias and social desirability bias, likely leading to an underestimation of the true prevalence. Moreover, the absence of quantitative exposure data (e.g. number of cigarettes smoked per day) precluded dose-response analyses. Second, NHANES does not provide a clear definition of ‘poor physical health’, making it difficult to differentiate between somatization symptoms and organic diseases. Third, given the cross-sectional design, causality cannot be inferred from the observed associations. Fourth, participants with missing data were excluded from the analysis; if these data were not missing completely at random, selection bias may have been introduced. Fifth, the relatively small sample size of the MSDP-exposed group (n=183) limited the statistical power for subgroup analyses. Sixth, although the sample is nationally representative after applying sampling weights, generalizability to populations outside the United States remains uncertain. Finally, the absence of key covariate information, including adolescent smoking and alcohol use, parental history of mental illness, and parental smoking status during childhood and adolescence, may have resulted in residual confounding.

## CONCLUSIONS

In this cross-sectional study using NHANES 2007–2012 data, MSDP was associated with higher odds of anxiety and poor mental health among US adolescents aged 12–15 years. Causality cannot be inferred given the cross-sectional design. Future studies with prospective, quantitative exposure assessment and more robust designs, such as adoption studies, are needed to confirm these findings.

## Supplementary Material



## Data Availability

The dataset used for this study analysis can be found on the official website of the National Health and Nutrition Examination Survey (https://www.cdc.gov/nchs/nhanes/index.htm).
