# Supplementary material for: Maternal smoking during pregnancy in relation to adolescent anxiety and poor mental health: A cross-sectional study based on NHANES 2007–2012
Source: Tob Induc Dis. 2026 Sep 12;24:10.18332/tid/226577. doi: 10.18332/tid/226577 (PMC13584921; doi:10.18332/tid/226577)
Supplement: Supplementary file 1 [file TID-24-163-s1.pdf]

Tobacco Induced Diseases

Supplementary file

© 2026 Liu Y. et al.

DOI:
10.18332/tid/226577

The content has been provided by the author(s) and has not been reviewed, verified, or endorsed by European Publishing. It may not have undergone peer review. The views, opinions, and recommendations expressed are solely those of the author(s) and do not necessarily reflect the position of European Publishing. European Publishing accepts no responsibility or liability for any consequences arising from the use of, or reliance on, this content.

Supplementary file

Supplementary Figure S1. Prevalence of high anxiety and poor mental health among 1,445 U.S. adolescents aged 12–15 years from NHANES 2007–2012, stratified by maternal smoking during pregnancy (MSDP)

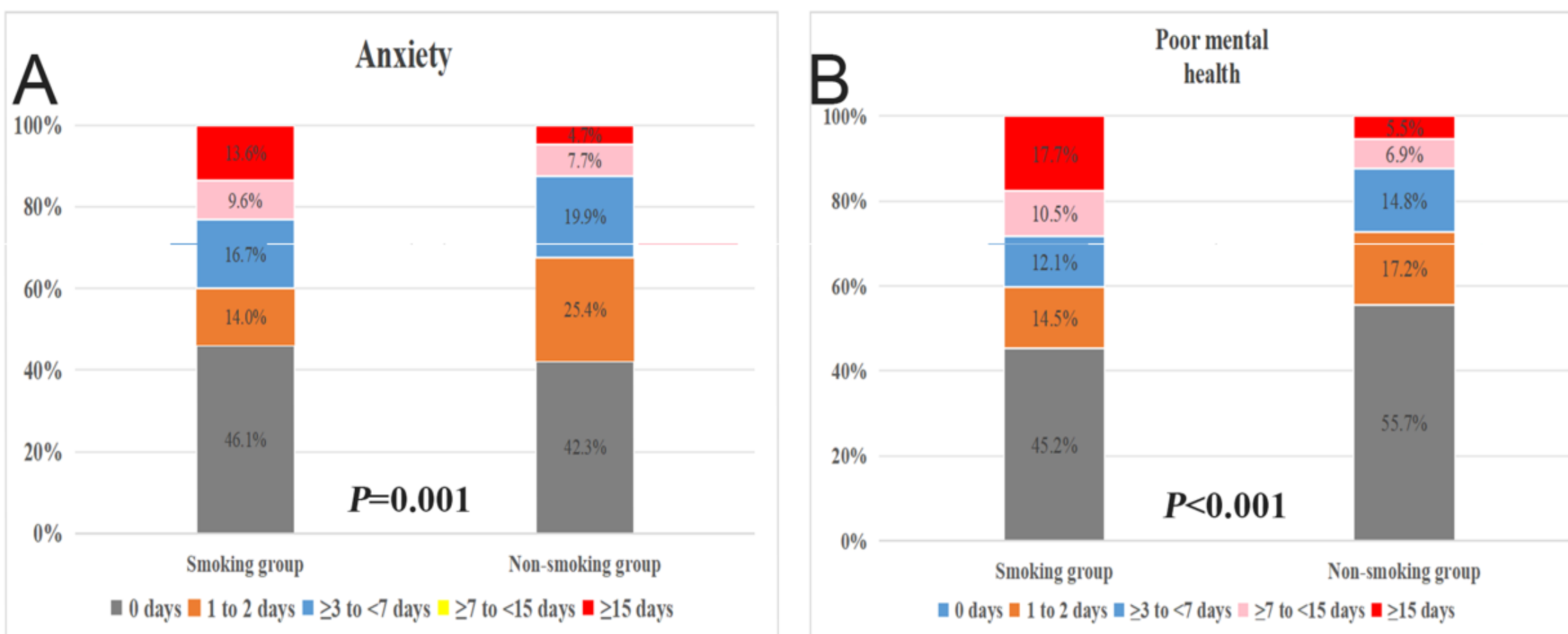

P-values were calculated using the Rao-Scott χ^2 test for categorical variables, accounting for the complex survey design.

Supplementary Table S1. Variance inflation factors (VIFs) for covariates in the fully adjusted models for anxiety and poor mental health outcomes among 1,445 U.S. adolescents aged 12–15 years (NHANES 2007–2012).

Characteristic	Df	High anxiety GVIF	High anxiety GVIF^(1/(2Df))	Poor mental health GVIF	Poor mental health GVIF^(1/(2Df))
Sex	1	1.04	1.02	1.05	1.03
Age	3	3.03	1.20	3.04	1.20
Race/ethnicity	2	1.44	1.10	1.42	1.09
Family PIR	2	1.61	1.13	1.57	1.12
BMI	2	1.07	1.02	1.08	1.02
Hypertension	1	1.05	1.03	1.05	1.02
Adolescent education level	2	2.93	1.31	2.94	1.31
Household size	1	1.23	1.11	1.22	1.11
Household head's education level	2	1.43	1.09	1.40	1.09
Parental marital status	1	1.27	1.13	1.28	1.13
Physical activity	2	1.09	1.02	1.10	1.03
MSDP	1	1.14	1.07	1.14	1.07
Poor physical health	1	1.03	1.01	1.03	1.01
Routine place to go for healthcare	1	1.16	1.08	1.16	1.08
Health insurance status	1	1.16	1.08	1.16	1.08
Low birth weight	1	1.03	1.02	1.04	1.02
Mother's age at child's birth	1	1.18	1.09	1.16	1.08

Multicollinearity was assessed using variance inflation factors (VIF) based on an unweighted logistic regression model. For categorical variables with more than two levels, the generalized variance inflation factor (GVIF) was calculated and adjusted to an equivalent VIF using the formula GVIF^(1/(2×Df)), where Df is the number of dummy variables for that predictor. Abbreviations: VIF, variance inflation factor; GVIF, generalized variance inflation factor; Df, degrees of freedom; PIR, poverty income ratio; BMI, body mass index; MSDP, maternal smoking during pregnancy.
